# The gut microbiota-bile acid axis in cholestatic liver disease

**DOI:** 10.1186/s10020-024-00830-x

**Published:** 2024-07-19

**Authors:** Dayan sun, Chuanping Xie, Yong Zhao, Junmin Liao, Shuangshuang Li, Yanan Zhang, Dingding Wang, Kaiyun Hua, Yichao Gu, Jingbin Du, Guoxian Huang, Jinshi Huang

**Affiliations:** 1 Department of Neonatal Surgery, Beijing Children’s Hospital, Capital Medical University, National Center for Children’s Health, No. 56 Nalishi Road, Xicheng District, Beijing, 100045 China; 2 Department of Pediatric Surgery, Women and Children’s Hospital, School of Medicine, Xiamen University, Xiamen, Fujian 361000 China

**Keywords:** Cholestatic liver disease, Gut microbiota-bile acids, Liver fibrosis, Microbial translocation, Therapeutic targets

## Abstract

Cholestatic liver diseases (CLD) are characterized by impaired normal bile flow, culminating in excessive accumulation of toxic bile acids. The majority of patients with CLD ultimately progress to liver cirrhosis and hepatic failure, necessitating liver transplantation due to the lack of effective treatment. Recent investigations have underscored the pivotal role of the gut microbiota-bile acid axis in the progression of hepatic fibrosis via various pathways. The obstruction of bile drainage can induce gut microbiota dysbiosis and disrupt the intestinal mucosal barrier, leading to bacteria translocation. The microbial translocation activates the immune response and promotes liver fibrosis progression. The identification of therapeutic targets for modulating the gut microbiota-bile acid axis represents a promising strategy to ameliorate or perhaps reverse liver fibrosis in CLD. This review focuses on the mechanisms in the gut microbiota-bile acids axis in CLD and highlights potential therapeutic targets, aiming to lay a foundation for innovative treatment approaches.

## Introduction

The gastrointestinal tract constitutes the largest mucosal surface within human bodies (de Vos et al. 2022). An intricate assembly of diverse cell types of epithelial and endothelial origin and innate and adaptive immunity system is located in the gastrointestinal mucosal barrier, empowering the body to promptly confront challenges from the complex environment (de Vos et al. 2022; Lee et al. 2022; Martel et al. 2022). The portal vein gathers blood from the small and large intestine, spleen, and pancreas and directs it into the liver, establishing a crucial connection between the liver and the intestines (Tilg et al. 2022). In healthy individuals, the liver and its immune system can transform potentially pathogenic or toxic compounds from the gastrointestinal tract into lower toxic metabolites, thereby maintaining the host’s overall health (Guilliams et al. 2022). In turn, liver secretion through the biliary tree into the intestine improves intestinal flora diversity and mucosal barrier integrity, establishing bidirectional crosstalk along the gut-liver axis (Wahlstrom et al. 2016). Disruptions within the gut microbiota-bile acid axis have been recognized for their role in exacerbating the progression of hepatic fibrosis across a spectrum of hepatic pathologies, such as non-alcoholic or alcoholic fatty liver disease, cholestatic liver diseases (CLD), hepatocellular carcinoma, and cholangiocarcinoma (Fuchs and Trauner 2022). This study provides a comprehensive overview of the mechanistic role of the gut microbiota-bile acid in the progression of CLD.

Cholestatic liver diseases emerge due to various intrahepatic and extrahepatic factors impeding bile formation, secretion, or excretion, which results in the accumulation of bile acids (BAs) within the liver and an elevated concentration of BAs in the circulatory system (Chiang and Ferrell 2020). The most common types of CLD include primary biliary cholangitis (PBC), primary sclerosing cholangitis (PSC), biliary atresia, and progressive familial intrahepatic cholestasis (PFIC) (Li et al. 2017). In the early stages of certain CLD, the clinical manifestations might be asymptomatic, with only alkaline phosphatase (ALP) and gamma-glutamyl transpeptidase (GGT) elevating (Shi et al. 2022). Nevertheless, as the disease progresses, hyperbilirubinemia might develop, culminating in liver fibrosis, cirrhosis, and failure, necessitating eventual liver transplantation (Chen et al. 2018; Zeng et al. 2023). Over the past two decades, a global surge in the incidence and prevalence of CLD has been observed. However, the development of efficacious therapeutic strategies to mitigate CLD progression remains a formidable challenge (Zeng et al. 2023).

There is growing attention on the disruption of the gut microbiota-bile acid axis as a culprit of liver fibrosis in CLD (Albillos et al. 2020; Zhang et al. 2022). Studies have demonstrated that under conditions of cholestasis, the impediment to bile drainage impairs the intestinal mucosal barrier, leading to bacteria translocation (Li et al. 2018; Yang et al. 2020). Gut microbiota can also modify the BAs pool, perturbing BAs synthesis and metabolism (Guzior and Quinn 2021). The interplay between the gut microbiota and BAs is bidirectional (Gijbels et al. 2021). Improving the detrimental loop of the gut microbiota-bile acid axis presents an innovative therapeutic strategy for CLD (Li et al. 2017; Laue and Baumann 2022). This review will provide a comprehensive summary of the gut microbiota-bile acid axis in the progression of CLD and present potential therapeutic approaches to ameliorate CLD progression by targeting this axis.

## Advances in the regulation of gut microbiota in CLD

The gut microbiota, characterized as a large and diverse community with trillions of microorganisms, colonizes the gastrointestinal tract, spanning from the oral cavity to the rectum (Tilg et al. 2022). In healthy individuals, the gut microbiota’s vast array of microbial species contributes to the resilience and stability of the ecosystem, providing the host with greater resilience to environmental challenges and pathogenic invasion. Gut microbiota dysbiosis is involved in liver fibrosis progression in CLD through multiple mechanisms, such as inflammation, immune activation, and metabolic disturbances (Tilg et al. 2022; Fuchs and Trauner 2022; Li et al. 2018; Wang et al. 2019).

### Alteration of gut microbiota in CLD

A series of studies have shown that cholestasis condition led to a reduction in gut microbiota diversity, an increase in potentially pathogenic bacteria, and a decrease in beneficial bacteria (Yang et al. 2020). Lynch et al. (2023) observed a robust positive correlation between the post-menstrual age and the variability in gut microbiome composition in neonates without cholestatic conditions. In contrast, no such correlation was evident in neonates with cholestasis, suggesting an impaired developmental trajectory of the gut microbiome in cholestasis status. Furthermore, they identified that *Clostridium perfringens* were depleted in stool from cholestatic neonates, leading to impaired deconjugation of BAs (Lynch et al. 2023).

Several studies have observed that the gut microbial composition altered in CLD, such as biliary atresia, primary sclerosing cholangitis (PSC), and primary biliary cholangitis (PBC) (Kummen and Hov 2019; Rager and Zeng 2023). Interestingly, the alterations in gut microbiota in CLD have some similarities, such as the increase in the abundance of *Streptococcus* and *Veillonella* genera, and the decrease in the abundance of *Faecalibacterium* (Thibaut and Bindels 2022; Wang et al. 2022). However, as shown in Table 1, there are also certain variations at the phylum or genus level among different types of CLD (Bajer et al. 2017; Lv et al. 2016; Ostadmohammadi et al. 2021). Biliary atresia, characterized as progressive and fibrotic biliary obstruction, is one of the most devastating hepatobiliary diseases in the neonatal period (Harpavat et al. 2020). In a comparative analysis by Wang et al. (2019) involving 34 patients with biliary atresia and 34 healthy controls (HC), a significant disparity in gut microbiota diversity and composition was observed. Patients with biliary atresia exhibited lower diversity and significant structural segregation compared to HC. At the phylum level, there was an increase in the abundance of *Proteobacteria* and a reduction in *Bacteroidetes* in biliary atresia. At the genus level, some potential bacteria, such as *Streptococcus* and *Klebsiella*, thrived in biliary atresia, while beneficial bacteria, such as *Bifidobacterium* and several butyrate-producing bacteria, diminished. In addition, the abundance ratio of *Streptococcus*/*Bacteroides* displayed great promise for diagnosing biliary atresia. Song et al. (2021) found *Streptococcus*, *Klebsiella*, *Veillonella*, and *Enterococcus* were dominant bacteria in biliary atresia. *Klebsiella* and *Veillonella* were closely associated with elevated liver enzymes (*p* < 0.05), while *Enterococcus* positively correlated with lithocholic acid derivatives (*p* < 0.05). Yang et al. (2022) reported that post-Kasai jaundice clearance was associated with *Campylobacter* and *Rikenellaceae* (*p* < 0.05). This implies that the alteration of gut microbiota can serve as a diagnostic tool for biliary atresia and a prognostic indicator for predicting long-term outcomes.

**Table 1:** Alterations of gut microbiota in cholestatic liver disease

Type of liver disease	Groups compared	Methodology	Results (vs. HC, at genus level)		Reference
			Increased	Reduced	
Biliary atresia	16 HC vs. 32 biliary atresia	Metagenomic sequencing Stool sample	Klebsiella, Streptococcus, Veillonella. Enterococcus	Bifidobacterium, Blautia	Song et al. (2021)
Biliary atresia	34 HC vs. 34 biliary atresia	16 S rRNA gene sequencing and metagenomic sequencing	Streptococcus, Klebsiella	Bifidobacterium, Faecalibacterium	Wang et al. (2019)
Biliary atresia	38 HC vs. 46 biliary atresia	16 S rRNA gene sequencing	Escherichia-Shigella, Streptococcus, Veillonella	Faecalibacterium, Actinomyces, Agathobacter, Blautia, Eggerthella	Yang et al. (2022)
PSC	8 HC; 12 IBD; 14 PSC-IBD	16 S rRNA gene sequencing	Unknown genus (Enterobacteriaceae family)	vs. HC: Bacteroidetes;	Ostadmohammadi et al. (2021)
PSC	263 HC; 36UC; 85 PSC	16 S rRNA gene sequencing	Veillonella	Coprococcus, Succinivibrio, Desulfovibrio, Phasocolarctobacterium, seven other unknown genus	Kummen et al. (2017)
PSC	52 HC; 52PSC; 13 UC; 30 CD	16 S rRNA gene sequencing	Enterococcus, Fuscobacterium, Lactobacillus, Morganella, Streptococcus	Anaerostipes	Sabino et al., 2017
PSC	31 HC; 43PSC; 32UC	16 S rRNA gene sequencing	Veillonella, Streptococcus, Enterococcus, Clostridium, Haemophilus, Rothia	Coprococcus, unknown genus (Lachnospiraceae family)	Bajer et al. (2017)
PSC	30 HC; 33 IBD; 49 PSC	16 S rRNA and ITS2 DNA gene sequencing	Veillonella	Ruminococcus, Ruminiclotridium, Faecalibacterium, Lachnoclostridium, Blautia	Lemoinne et al. (2019)
PBC	80 HC; 60 PBC	16 S rRNA gene sequencing	Haemophilus, Veilonella, Clostridium, Lactobacillus, Streptococcus, Pseudomonas, Klebsiella, Unknown genus (Enterobacteriaceae)	Bacteroidetes spp, Sutterella, Oscillospira, Faecalibacterium	Tang et al. (2018)
PBC	30 HC; 42 PBC	16 S rRNA gene sequencing	Bifidobacterium, Veillonella, Neisseria, Klebsiella	Desulfovibrio, Megamonas	Lv et al. (2016)

PSC: primary sclerosing cholangitis; PBC: primary biliary cholangitis; HC: healthy control group

So far, numerous high-quality studies have identified alterations in gut microbiota in PSC and PBC (Abe et al. 2018; Lemoinne et al. 2019). Compared to PBC, PSC shows a strong association with inflammatory bowel disease (IBD) and is considered more representative of typical gut-liver axis disorder (Kummen and Hov 2019). Although there might be variations in the specific microbial change observed across different studies on PSC, they consistently reveal distinctions in gut microbial composition between PSC/PSC-IBD patients and HC or IBD patients. These distinctions include the following aspects: (a) a reduction in bacterial diversity and diminished bacterial richness; (b) an enrichment of pathogenic bacterial genera in PSC patients, such as *Streptococcus*, *Veillonella*, and *Enterococcus*. For instance, Kummen et al. (2017) found a 4.8-fold increase of *Veillonella* in PSC compared to HC, and it could be applied as a promising predictive marker for diagnosing PSC. Sabino et al. (2016) demonstrated a positive correlation between *Enterococcus* and elevated levels of alkaline phosphatase (ALP) (*p* = 0.048), a clinical marker for cholestasis; (c) the presence of IBD has a negligible effect on the composition of gut microbiota (Sabino et al. 2016). In addition, Tang et al. (2018) observed a significant reduction in gut microbiota diversity in PBC compared to HC. Importantly, the alterations in gut microbiota can distinguish PBC from HC (area under curve = 0.86, 0.84 in exploration and validation area, respectively). Subsequent research revealed that treatment with ursodeoxycholic acid (UDCA) in PBC patients partially alleviated the dysbiosis of gut microbiota, suggesting that gut microbiota could become a potential therapeutic target for PBC (Tang et al. 2018).

In summary, it is reasonable to infer significant alternations in gut microbiota in CLD, and the specificity of alterations in gut microbiota might become a new diagnostic tool for CLD (Tilg et al. 2022). Besides, the intervention of gut microbiota might become a potential therapeutic target for CLD (Guzior and Quinn 2021). However, there are still some problems to be solved. For example, despite the apparent overlapping microbial features in the microbiomes of patients with CLD, suggesting certain similarities in CLD, such as changes in *Streptococcus* and *Veillonella* genera in PSC/PBC/biliary atresia, the etiology of these similar microbial changes remains unclear. Therefore, further investigation is required to explore the role of gut microbiota in the pathogenesis of CLD.

### The effect of gut microbiota dysbiosis on liver fibrosis in CLD

Despite alterations in gut microbiota in CLD, the specific mechanisms of how gut microbiota dysbiosis promotes liver fibrosis are still under exploration. Zhou et al. (2023) confirmed that the depletion of gut microbiota exacerbated cholestatic liver injury in mice under bile duct ligation (BDL). The elevated plasma ALT and ALP levels are associated with diminished gut microbiota diversity and increased Gram-negative bacteria, suggesting the significant role of gut microbiota in the progression of CLD (Schneider et al. 2021). Gut microbiota dysbiosis promotes liver fibrosis progression through multiple mechanisms, including disturbances in the intestinal mucosal barrier, immune response, and metabolic disorders (Tilg et al. 2022; Zhang et al. 2022). Intestinal mucosal barrier dysfunction and increased intestinal permeability provided channels for the entry of gut microbiota and metabolites into the liver (Tilg et al. 2022; Qi et al. 2020). Subsequently, the gut microbiota and their metabolites can act as messages to activate hepatic inflammatory signaling pathways, leading to liver fibrosis progression (Qin et al. 2014).

**Fig. 1:**
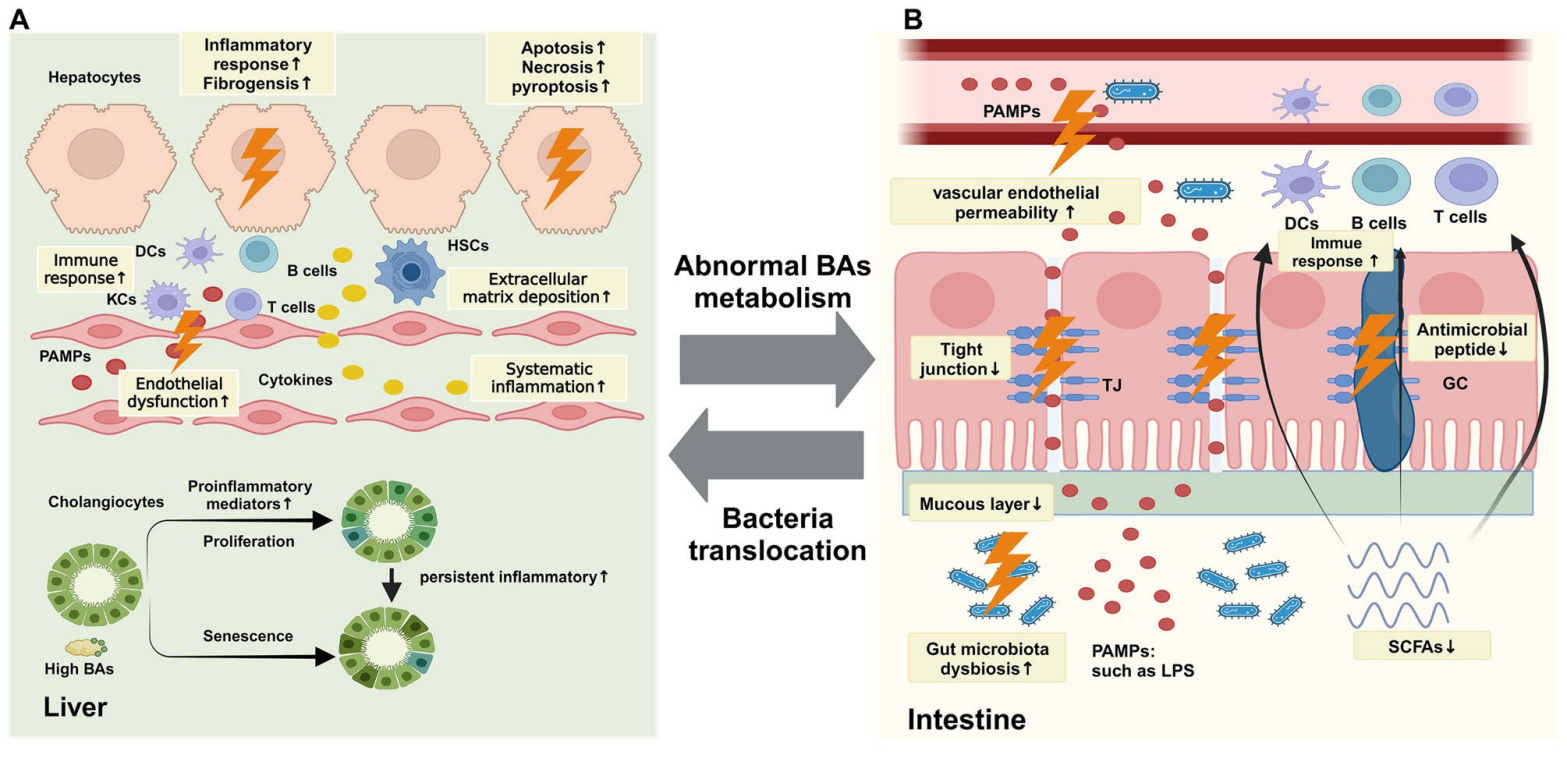
Abnormal gut microbiota-bile acids in CLD. (**A**) Abnormal BAs signaling promotes the progression of CLD, and the mechanisms are as follows: (i) the aviation of immune response associated with released numerous pro-inflammatory signals and increased shedding of inflammatory mediators, including cytokines, chemokines, and adhesion molecules, leading to systematic inflammation, (ii) the aviation of hepatic stellate cells (HSCs) leading to extracellular matrix (ECM) deposition, endothelial dysfunction of liver sinusoidal epithelial cells (LSECs), and fibrogenesis formation, (iii) Induction of hepatocytes death through multiple death pathways, including apoptosis, necrosis, and pyroptosis, (iv) gut bacteria and pathogen-associated molecular patterns (PAMPs) enter the liver through the portal vein and promote immune responses, (v) the disruption of tight junctions between cholangiocytes leading to inflammatory and fibrotic response, and cholangiocytes proliferation and senescence. (**B**) Bacterial translocation participates in liver fibrosis progression in CLD, comprising (i) gut microbiota dysbiosis, (ii) reduced excretion of mucosal thickness and antimicrobial peptide, (iii) disrupted tight junctions between intestinal epithelial cells, (iv) vascular endothelial permeability, (v) the decreasing synthesis of short-chain fatty acids (SCFAs), which enabled the disruption of intestinal mucosal barrier and translocation of bacteria and pathogen-associated molecular patterns (PAMPs) into the liver through portal vein. BA, bile acids; DCs, dendritic cells; TJ, tight junction; KC, Kupffer cells; HSCs, hepatic stellate cells; PAMPs, pathogen-associated molecular patterns; LPS, lipopolysaccharide; SCFAs, short-chain fatty acids;

The intestinal mucosal barrier serves as the primary defensive interface against the translocation of luminal contents into the hepatic circulation (Fig. 1). It mainly consists of gut microbiota, intestinal mucus layer, and intestinal epithelial cells (Chopyk and Grakoui 2020). The integrity of the intestinal mucosal barrier is crucial to preventing microbial translocation (Schnabl and Brenner 2014). Gut microbiota plays a vital role in the intestinal mucosal barrier function through various mechanisms (Zhou et al. 2023; Schnabl and Brenner 2014). Firstly, gut microbiota dysbiosis disrupts tight junctions between intestinal epithelial cells, inhibits mucus production, and reduces antimicrobial peptide release (Hiippala et al. 2018; Wells et al. 2017). Tight junctions are integral components between adjacent intestinal epithelial cells, controlling the transport across the epithelium and intestinal epithelial cell permeability (Tilg et al. 2022; Albillos et al. 2020; Turner 2009). Sorriba et al. (2019) have reported that, in comparison to healthy controls (HC), there is a reduction in the ileal epithelial mucosal thickness, loss of goblet cells, bacterial overgrowth, and increased vascular endothelial permeability, all of which contribute to an impaired intestinal epithelial barrier. Once the intestinal mucosal barrier is disrupted, gut bacteria and pathogen-associated molecular patterns (PAMPs) such as lipopolysaccharide (LPS), lipoteichoic acid (LTA), peptidoglycans, endotoxins, can penetrate the intestinal mucosal barrier and enter the liver through the portal vein, inducing innate immune responses activated by Toll-like receptors (TLRs) and nucleotide-binding oligomerization domain-like receptors (NLRs), as well as adaptive immune responses mediated by T cells and B cells, promoting liver fibrosis progression (Wang et al. 2022; Mridha et al. 2017; Vaishnava et al. 2011). The TLR family consists of transmembrane proteins in hepatic cells that recognize gut microbiota and their metabolites, activating the innate immune system. Among them, TLR4 has been studied extensively, and PAMPs such as LPS can bind to TLR4, activating the NF-κB signaling pathway, upregulating inflammatory factors such as TNF-α, IL-1β, and IL-6, inducing hepatic stellate cells (HSCs) activation, and promoting extracellular matrix synthesis, leading to liver fibrosis progression (Mridha et al. 2017). NLRs are a class of pattern recognition receptors that can induce the expression of NOD-like receptor protein 3 (NLRP3) in the cytoplasm. NLRP3 can recognize pathogenic substances from damaged cells and activate the caspase-1 signaling pathway through the innate immune pathway, which can produce IL-1 and IL-18 and stimulate the aviation of hepatic stellate cells (Assimakopoulos and Charonis 2013; Liao et al. 2019; Wree et al. 2014). Furthermore, after entering the portal circulation, gut microbiota and their metabolites can be captured by antigen-presenting cells, activating adaptive immune responses, such as T and B cells, regulating hepatic stellate cell activation, and promoting liver fibrosis progression (Tilg et al. 2022; Hammerich et al. 2014; Novobrantseva et al. 2005).

Dysbiosis of gut microbiota can also disrupt microbial metabolism, resulting in decreased short-chain fatty acids (SCFAs) synthesis. Jee et al. (2022) found that feeding with butyrate salts in BDL mice could alleviate the progression of liver fibrosis and improve the survival of biliary epithelial cells. SSCFAs, predominantly derived from the fermentation of dietary fibers by the gut microbiota, include acetate, butyrate, propionate, and formate, which collectively account for 90-95% of the SCFA pool (Rios-Covian et al. 2016). SCFAs not only act as an energy source for intestinal epithelial cells, but also regulate immune cells, maintaining the integrity of the intestinal epithelial mucosal barrier (Kim 2018). Acetate can bind to surface receptors of dendritic cells (DCs), inducing the release of infection-free globules (IgA) through B cells aviation. Propionate and butyrate can inhibit dendritic cells (DCs) CD40 expression, reducing IL-6 and IL-12 expression (Nastasi et al. 2015). Butyrate can also regulate Treg cell differentiation through dendritic cells to modulate immune responses (Singh et al. 2014). Treg cells are a CD4 + T cell subset that serves as the only negative regulator of immune cells in the body, inhibiting the proliferation of Th cells (such as Th1, Th2, and Th17 cells) and thereby suppressing inflammatory responses (Dong et al. 2015). In conclusion, the dysbiosis of gut microbiota promotes the progression of liver fibrosis through multiple pathways, such as disruption of the intestinal mucosal barrier, immune response, and decreased synthesis of SCFAs. Maintaining gut microbiota homeostasis plays a vital role in improving the progression of liver fibrosis (Zhang et al. 2022; Juanola et al. 2021).

## Role of bile acids in the CLD progress

### BAs homeostasis

**Fig. 2:**
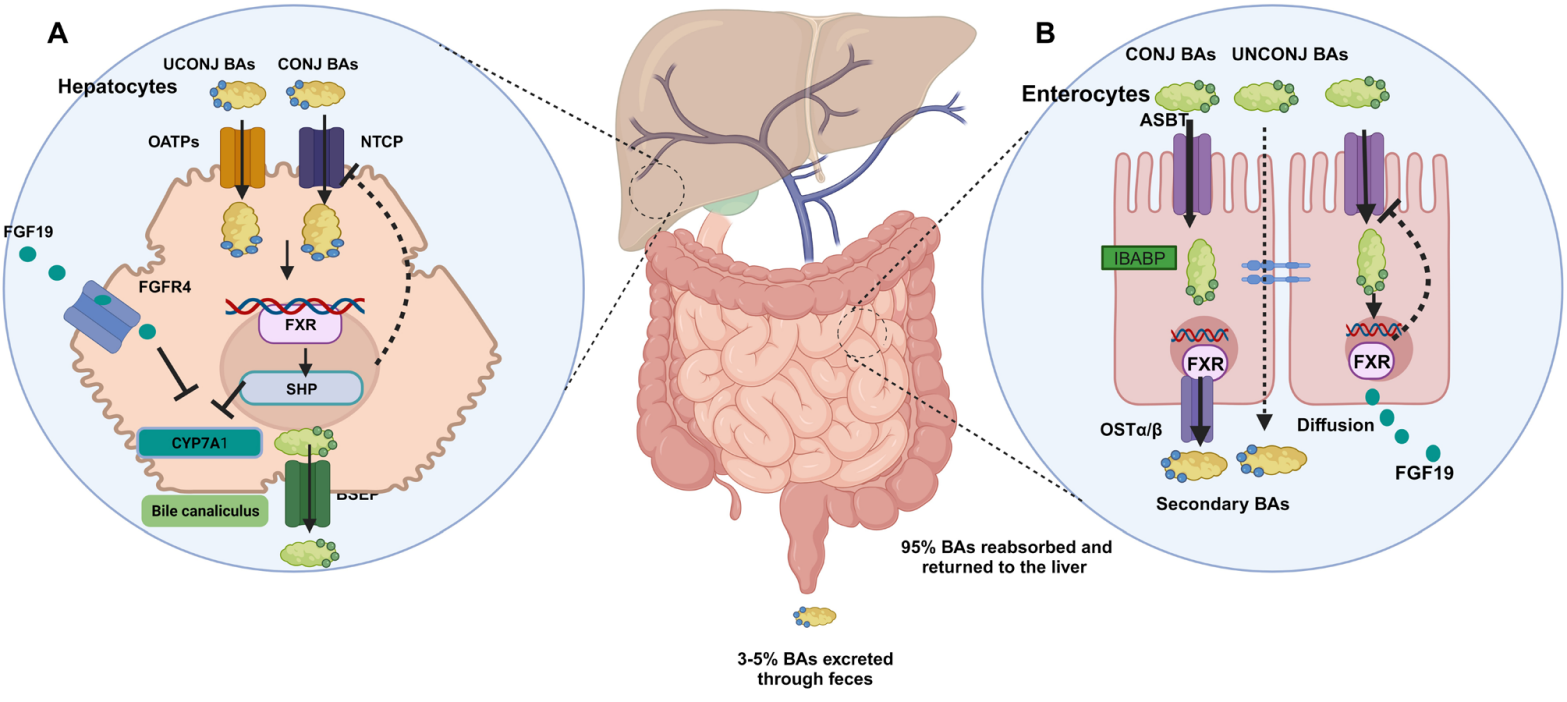
Bile acids transport and signaling along the gut-liver axis. (**A**) Hepatocellular BA homeostasis. Most conjugated BAs are reabsorbed via sodium taurocholate co-transporting polypeptide (NTCP) and unconjugated BAs through organic anion-transporting polypeptides (OATPs). BAs are taken up from the portal vein and synthesized by two main enzymatic processes: the classical pathway, predominantly initiated by cholesterol 7α-hydroxylase (CYP7A1) (responsible for the majority of bile acid synthesis), and the alternative pathway, mediated by sterol 27-hydrolase (CYP27A1) (not shown here), which are excreted into bile canaliculi via the bile salt export pump (BSEP). In addition, the FXR signaling pathway (activated by BAs) can inhibit CYP7A1 and CYP8B1 gene expression, suppress NTCP expression, and induce BSEP expression, thus protecting hepatocytes from BAs toxicity. BAs synthesis is also inhibited by fibroblast growth factor 19 (FGF19), which is transported back to the liver via fibroblast growth factor receptor 4 (FGFR4) and suppresses the activity of CYP7A1. (**B**) In the terminal ileum, primary conjugated BAs are actively taken up into enterocytes via the apical sodium-dependent BA transporter (ASBT), transported through the basolateral membrane by the ileal bile acid-binding protein (IBABP), and enter the portal vein via the organic solute transporter-α/β (OSTα/β). Unconjugated BAs can be taken up by passive diffusion through the intestine. Intestinal BAs enhance the expression of the FXR/FGF19 signaling pathway, which is transported back to the liver and suppresses BA synthesis. In addition, the aviation of FXR signaling downregulates ASBT expression and induces OSTα/β expression, protecting enterocytes from BAs toxicity. NTCP, sodium taurocholate co-transporting polypeptide; OATPs, organic anion-transporting polypeptides; FGF19, fibroblast growth factor 19; FGFR4, fibroblast growth factor receptor 4; ASBT, apical sodium-dependent BA transporter; IBABP, ileal bile acid-binding protein; CYP7A1, cholesterol 7α-hydroxylase

BAs are primarily synthesized from cholesterol, with the synthesis pathways involving two main enzymatic processes: the classical pathway, predominantly initiated by cholesterol 7α-hydroxylase (CYP7A1) (responsible for the majority of bile acid synthesis), and the alternative pathway, mediated by sterol 27-hydrolase (CYP27A1) (accounting for approximately 10% of total bile acid synthesis) (Zeng et al. 2023). These pathways yield two free primary BAs: cholic acid (CA) and chenodeoxycholic acid (CDCA) (Chiang 2004). Irrespective of the initial pathway, the last step in BA synthesis involves the conjugation of primary BAs with glycine or taurine, which is facilitated by bile acid coenzyme A amino acid N-acetyltransferase (BAAT). This enzyme-catalyzed reaction maintains the amphipathic structure and allows BAs to be impermeable through the membrane (Dawson et al. 2009; Monte et al. 2009). Subsequently, the majority of BAs are actively transported into bile canaliculi via the bile salt export pump (BSEP). In the terminal ileum, primary conjugated BAs undergo microbial enzymatic conversion and are converted into secondary BAs, such as deoxycholic acid (DCA), lithocholic acid (LCA), and ursodeoxycholic acid (UDCA). The majority of conjugated BAs are actively taken up into enterocytes via the apical sodium-dependent BA transporter (ASBT), transported through the basolateral membrane by the ileal BA-binding protein (IBABP), and then enter the portal vein via the organic solute transporter-α/β (OSTα/β). Unconjugated BAs can be taken up by passive diffusion through the intestine. In the liver, most BAs are reabsorbed into hepatocytes via sodium taurocholate co-transporting polypeptide (NTCP). A minor fraction of unconjugated BAs returned to hepatocytes through organic anion-transporting polypeptides (OATPs) (Zhang et al. 2022). Approximately 95% of BAs are reabsorbed into the liver by the gut-liver axis, while the remaining unabsorbed portion is excreted with feces and urine (Fig. 2) (Guzior and Quinn 2021).

The synthesis and transport of BAs are also regulated by a sophisticated network of hormone-like signaling (Chiang 2009; Lu et al. 2000). BAs can bind and activate members of the cell surface and nuclear receptors family, collectively referred to as BA-activated receptors (Fiorucci et al. 2020). Key receptors include the Farnesoid X receptor (FXR), Takeda G protein-coupled receptor 5 (TGR5), Liver X receptor (LXR), and sphingosine-1-phosphate receptor 2 (S1PR2) (Wan and Sheng 2018). Among these receptors, FXR is the most crucial receptor that regulates BA homeostasis (Yan et al. 2021). FXR signaling regulates BAs synthesis through two principal pathways (Fig. 2). Firstly, the FXR signaling pathway regulates the gene expression of crucial enzymes involved in BAs synthesis (Goodwin et al. 2000). FXR aviation promotes the expression of the target gene SHP, leading to the downregulation of CYP7A1 and CYP8B1 gene expression (Xiang et al. 2021). Notably, loss of SHP in mice fed BAs showed that inhibition of CYP7A1 gene expression was retained, indicating the presence of compensatory repression pathways of BA signaling independent of SHP (Kerr et al. 2002; Wang et al. 2002). Moreover, intestinal BAs aviation enhances the expression of FGF15/19, which is transported back to the liver via the portal vein system, activating the JNK signaling pathway by binding with fibroblast growth factor receptor 4 (FGFR4), thereby inhibiting CYP7A1 expression (Li et al. 2017). Additionally, studies revealed that the expression of FGF19 in the liver tissue of patients with biliary atresia or PBC was significantly higher than FGF19 in serum, suggesting that intrahepatic auto-/paracrine FGF19 feedback signaling might exist (Al-Khaifi et al. 2018; Nyholm et al. 2023).

Secondly, FXR signaling also plays a pivotal role in regulating bile acid transporters. Experimental studies demonstrated that the aviation of the FXR/SHP pathway downregulates ASBT expression in the ileum (Chen et al. 2003; Neimark et al. 2004). The subsequent suppression of ASBT has been associated with a reduction in the expression of pro-fibrogenic genes, thereby attenuating the progression of liver fibrosis (Baghdasaryan et al. 2016; Miethke et al. 2016). FXR aviation also induced OSTα/β expression, which protected enterocytes from BA toxicity. Elevated OSTα/β expression has been observed in BDL mice as well as patients with PBC. The upregulation of OSTα/β might serve as a baolateral “pressure relieve valve” for consistently elevated intracellular BA concentration (Boyer et al. 2006; Gulamhusein and Hirschfield 2020). In addition, the FXR/SHP signaling pathway represses the expression of NTCP in hepatocytes in BDL mice, which protects hepatocytes from BA toxicity (Geier et al. 2005; Robin et al. 2018; Zollner et al. 2002). NTCP inhibitor is promising to become a novel therapy for HBV/HDV and has demonstrated liver protection in cholestatic mice (Bogomolov et al. 2016; Slijepcevic et al. 2018). FXR aviation could induce BSEP expression, facilitating BA secretion into bile canaliculi (Ananthanarayanan et al. 2001; Plass et al. 2002). Inherited BSEP deficiency leads to cholestasis in humans, underscoring the critical role of BSEP in BA synthesis (Fuchs et al. 2017).

### Changes in the BAs profile in CLD progress

Based on their concentration, BAs might exhibit either beneficial or cytotoxic effects in hepatic and gastrointestinal diseases (Jansen et al. 2017). BAs at physiological concentrations can not only facilitate the metabolism of fats and cholesterols but also enhance immune response and maintain the balance of gut microbiota (Tilg et al. 2022). However, excessive levels of BAs may trigger inflammatory pathways and kill hepatocytes (Fuchs and Trauner 2022). BAs toxicity might not only be dependent on their concentration but also on their hydrophobicity and conjugation status, with the following increasing order of toxicity: UDCA< CA< CDCA< DCA< LCA (Hegade et al. 2016). BAs with greater hydrophobicity are more prone to penetrate cell membranes, thus disrupting intracellular structures and processes, leading to cellular damage and toxicity (Fuchs and Trauner 2022; Zeng et al. 2023). Hydrophobic BAs have been implicated in liver fibrosis through various pathways (Fig. 2). They can initiate inflammation response by stimulating the production of mediators, such as cytokines, chemokines, and adhesion molecules (Allen et al. 2011). Studies have demonstrated that hydrophobic BAs could induce the hepatocellular expression of adhesion factors and neutrophil chemoattractants, including ICAM1, CXCL1, CXCL2, and IL-17, leading to neutrophil aggregation (Fuchs and Trauner 2022). Additionally, BAs can promote the expression of the transcription factor early growth factor (EGR1), releasing pro-inflammatory signals in hepatocytes (Allen et al. 2010). The activation of EGR1-dependent pro-inflammatory signaling can induce the expression of IL-23 in hepatocytes, which is integral to the infiltration and activation of T helper 17 (Th17) cells (Licata et al. 2013; O’Brien et al. 2013). Hepatic accumulation of BAs converts DCs into an immunogenic myeloid phenotype with an enhanced ability to prime allogeneic and syngeneic T lymphocytes and secretes pro-inflammatory cytokines (Carambia et al. 2014; Rahman and Aloman 2013). Macrophage necrosis plays a pivotal role in liver fibrosis. In cholestatic conditions, conjugated BA glycodeoxycholate (GDCA) upregulates the Z-DNA-binging protein 1 (ZBP1)/phosphorylated-mixed lineage kinase domain-like pseudokinase (p-MLKL) signaling pathway and mediates macrophage necrosis, exacerbating liver fibrosis progression (Yang et al. 2023).

High BAs concentrations can induce cell death through multiple pathways in CLD, including apoptosis, necrosis, and pyroptosis (Jansen et al. 2017; Chen et al. 2001). Firstly, Hydrophobic BAs can activate mitochondrial oxidative stress, leading to alterations in mitochondrial permeability, the release of cytochrome C, and the initiation of a caspase cascade reaction, culminating in hepatocyte apoptosis (Lemasters et al. 1998; Yin and Ding 2003). Additionally, BAs can activate death receptors through an exogenous pathway, facilitating the formation of death receptor signaling complexes, activation of Caspase 8, and translocation of pro-apoptotic proteins to the mitochondria, leading to irreversible cell death (Faubion et al. 1999; Malhi and Kaufman 2011). Secondly, hydrophobic BAs can trigger necrosis through oxidative-stress-induced lipid peroxidation and dissolution of the hepatocellular plasma membrane (Shojaie et al. 2020). Afonso et al. (2016) found a high level of MLKL and receptor-interacting protein kinase (RIPK3) expression in patients diagnosed with PBC, confirming the presence of hepatic necrosis in CLD. Using the BDL animal model, further investigation showed a significantly increased pMLKL/MLKL after three days of ligation, suggesting that hepatocyte necrosis might represent an early event in the pathogenesis of CLD. Thirdly, in recent years, studies have revealed that pyroptosis might play a vital role in the progression of liver fibrosis. Gan et al. (2022; Xiao et al. 2023). Pyroptosis is a recently described programmed cell death with the release of numerous inflammatory cytokines (Gan et al. 2022; Yu et al. 2021). Chen et al. found BAs targeting mitofusin 2 (MFN2) differentially modulated innate immunity in physiological versus cholestatic states. BAs at a physiological concentration can promote mitochondrial fusion and enhance phagocytosis of macrophages, while BAs at high concentrations promote mitochondrial tethering to the endoplasmic reticulum, leading to mitochondrial calcium overload, which activates NLRP3 inflammasome and pyroptosis (Che et al. 2023). Xu et al. further analyzed the mechanisms of BAs-induced pyroptosis. Different from the LPS-induced GSDMD-dependent canonical pyroptosis pathway, BAs mainly aviated caspase-11 and GSDME-mediated non-canonical pyroptosis pathway underlies cholestatic liver damage (Xu et al. 2021).

Compared to elevated serum or hepatocellular BAs concentration, bile leakage from canaliculi and bile duct might play a more significant role in developing cholestatic liver injury (Jansen et al. 2017). High concentrations of BAs can disrupt tight junctions between biliary epithelial cells, resulting in bile leakage in the periductal region and the subsequent activation of inflammatory and fibrotic responses (Zeng et al. 2023). The accumulation of BAs can promote cholangiocyte proliferation and periportal fibrosis. Cholangiocyts proliferation, known as ductular reaction (DR), represents an adaptive response of cholangiocyts (Bertolini et al. 2022; Wang et al. 2017). Cholangiocyts often exhibit a neuroendocrine-like phenotype after DR, expressing numerous anti-apoptotic genes, adhesion molecules, cytokines, chemokines, and growth factors, which promotes the aviation, migration, and proliferation of myofibroblasts (Zeng et al. 2023). In BDL mice, DR can be observed, accompanied by the secretion of growth factors and inflammatory cytokines such as epidermal growth factor (EGF), vascular endothelial growth factor (VEGF), IL-6, and tumor necrosis factor (TNFα) (Munshi et al. 2011). While early DR-induced bile duct injury might regress, persistent BA concentration and inflammatory response can induce infiltration of the mesenchymal cell, promote periductal inflammatory response, induce cholangiocyte apoptosis or senescence, leading to the progression of portal fibrosis and even potential malignant transformation (Meadows et al. 2021; Yokoda and Rodriguez 2020). Consequently, further research is warranted to explore the critical pathways involved in DR in CLD.

BAs can also exert anti-inflammatory pathways via interacting with BA nuclear receptors and inhibiting immune responses (Fuchs and Trauner 2022; Wang et al. 2008). FXR is expressed in various immune cells. The aviation of FXR inhibits Toll-receptor 9 (TLR9)-dependent expression in macrophages and suppresses pro-inflammatory activity (Renga et al. 2013). FXR activation can also downregulate cytoplasmic NLRP3 expression, reducing inflammatory responses (Hao et al. 2017). FXR signaling pathway antagonizes NF-kB target gene expression in dendritic cells, including TNFα and inducible nitric oxide synthase (iNOS) (Wang et al. 2008). In natural killer T cells, FXR aviation suppresses the production of osteopontin, IL-1β, and IFN-γ (Gadaleta et al. 2011).

Takeda G protein-coupled receptor 5 (TGR5) is identified in macrophages as the first G-protein-linked receptor (GPCR) activated by BAs (Keitel et al. 2009). TGR5 exhibits high expression in non-parenchymal liver cells, including hepatic sinusoidal endothelial cells, hepatic stellate cells, and cholangiocytes (Reich et al. 2021). The aviation of TGR5 promotes intracellular bucolic adenosine monophosphate (cAMP) production and protein kinase A (PKA) aviation, thereby inhibiting NF-kB signal transduction and reducing inflammatory response (Guo et al. 2016; Keitel and Häussinger 2018). More importantly, TGR5 seems necessary for biliary tree development (Reich et al. 2016). TGR5 aviation promotes cholangiocyte regeneration, which is crucial for maintaining the integrity of the biliary tree and mitigating BA toxicity through stimulating bicarbonate secretion (Reich et al. 2021; Trauner and Fuchs 2022). TGR5-knockout BDL mice appeared more susceptible to cholestatic liver injury than wild-type mice (Reich et al. 2021; Pean et al. 2013).

The body employs compensatory defense mechanisms to alleviate the toxic effects of bile stasis in hepatic and gastrointestinal cells (Zollner et al. 2006). These mechanisms include adaptive changes in BAs receptors and activation of anti-inflammatory responses. However, these adaptations prove insufficient in countering the sustained liver damage induced by prolonged cholestasis. Moreover, cholestatic status can lead to downregulation and functional impairment of BA receptors and transport systems (Li et al. 2017). Consequently, further research is essential to investigate the pivotal pathways involved in liver fibrosis and bile duct injury, aiming to develop potential therapeutic strategies for mitigating the progression of CLD.

## The gut microbiota-bile acid crosstalk in CLD

### Bidirectional interactions between gut microbiota and BAs

#### Regulation of BAs profiles by gut microbiota

The intricate interplay of the gut microbiota encompasses a diverse array of mechanisms that govern the metabolism of BAs. The gut microbes are instrumental in the conversion of primary BAs into secondary BAs through multiple metabolic pathways, such as deconjugation, dehydroxylation, oxidation, isomerization, esterification, and desulfation (Li et al. 2017; Cai and Boyer 2021). These bioactive secondary BAs participate in the enterohepatic circulation, where they modulate the composition of BA pools and contribute to the maintenance of the host’s metabolic homeostasis (Zhang et al. 2022; Li et al. 2021). The initial phase of microbial BAs metabolism hinges on the enzymatic activity of bile salt hydrolase (BSH), which catalyzes the deconjugation reaction, hydrolyzing glycine- and taurine-conjugated BAs into their free forms (Li et al. 2017; Guzior and Quinn 2021). Gut microbiota exhibiting the BSH enzyme are varied and encompass multiple species, including *Bacteroides*, *Clostridium*, *Lactobacillus*, *Bifidobacterium*, *Enterococcus*, *Ruminococcaceae*, and *Listeria* (Chascsa et al. 2017). Lynch and colleagues reported a substantial reduction in BSH gene abundance and enzyme activity in neonates with bile stasis, leading to the impaired synthesis of unconjugated BAs synthesis and diminished gut microbiota diversity (Lynch et al. 2023). Further downstream, the gut microbiota facilitates unconjugated BAs dehydroxylation, oxidation, and isomerization processes (Guzior and Quinn 2021). Particularly noteworthy is the role of the Bai operon of *Clostridium* in orchestrating 7α-dehydroxylation, which transforms cholic acid (CA) into deoxycholic acid (DCA) and chenodeoxycholic acid (CDCA) into lithocholic acid (LCA) (Thibaut and Bindels 2022). Additional modifications enacted by bacteria encompass oxidation, epimerization, desulfation, and esterification (Zhang et al. 2022; Guzior and Quinn 2021). For instance, *Oxalobaceraceae*, *Enterobacteriaceae*, *Clostridiaceae*, *Escherichia*, and *Lachnospiraceae* contribute to BA oxidation and isomerization (Guzior and Quinn 2021). In parallel, *Oxalabacteraceae*, *Enterobacteriaceae*, and *Lactobacillus* are involved in bile acid esterification, and *Clostridiaceae*, *Stretociccaeae*, and *Pseudomonadaceae* are implicated in BA desulfation (Guzior and Quinn 2021; Jia et al. 2018; Just et al. 2018).

Given the potential for oxidation, epimerization, and dehydroxylation at multiple hydroxyl groups in BAs, the diversity of human bile acids extends beyond 2800 distinct variants (Guzior and Quinn 2021). This diversity exerts a pronounced influence on gut microbiota and the host. Alteration in oxidation and isomerization status modulate BA’s hydrophobicity and toxicity profiles, thereby protecting the hepatocytes and enterocytes from more hydrophobic and hazardous BAs (Guzior and Quinn 2021; Kisiela et al. 2012). In addition to stimulating the conversion from primary BA to secondary forms, the gut microbiota governs BA synthesis by regulating BA synthetic enzymes. Sayin and collaborators unveiled that gut microbiota could downregulate the expression of taurine-β-muri cholate sodium salt and upregulate FGF15 expression through FXR receptor aviation, thereby enhancing hepatic CYP7A1 expression and promoting BAs synthesis (Sayin et al. 2013). Moreover, investigations by Kim et al. confirmed that in the absence of gut microbiota, FXR antagonists remained unmetabolized, underscoring the potential role of the gut microbiota in the regulation of BAs synthesis via the FXR-FGF15/19 signal pathway (Schneider et al. 2021; Kim et al. 2007).

#### BAs as regulators of gut microbiota

BAs also assume a pivotal role in modulating the gut microbiota composition and upholding the integrity of the intestinal mucosal barrier (Albillos et al. 2020). Compromised integrity of this barrier can establish a conduit for intestinal bacteria and their metabolites to access the liver via the portal vein, thereby contributing to the progression of liver fibrosis (Tilg et al. 2022; Yang et al. 2020). In cholestatic conditions, the depletion of BAs within the intestinal lumen could trigger microbial dysbiosis and disruption of the intestinal epithelial mucosal barrier, culminating in microbial translocation (Li et al. 2017). Nevertheless, oral administration of BAs has demonstrated the capacity to mitigate excessive bacterial proliferation and microbial translocation in mice afflicted with liver cirrhosis (Simbrunner et al. 2021; Verbeke et al. 2015). Under normal physiological conditions, the intestinal mucosal barrier is meticulously regulated by intricate mechanisms that prevent the translocation of bacteria and PAMPs from the mucosal stratum (D’Aldebert et al. 2009). The occurrence of microbial translocation suggests a disruption of these mechanisms, including encompassing the downregulation of antimicrobial peptides, reduction in mucosal thickness, diminished expression of tight junction proteins, and impairment of intestinal vascular carriers (Xiang et al. 2021). Ongoing research underscores the potential of BAs metabolism to impinge upon the integrity of the intestinal mucosal barrier through the following mechanisms: Firstly, the deficiency of intestinal BAs could alter the composition of gut microbiota. BAs can stimulate the proliferation of intestinal flora, such as Enterococcus faecalis, Escherichia coli, and Listeria (Devkota and Chang 2015; Gahan and Hill 2014). Furthermore, previous studies have indicated that the absence of BAs could lead to a significant decline in enzyme activity and quantity of 7α-dehydroxylation, underscoring the indispensable role of BAs in fostering the growth of gut microbial communities (Guzior and Quinn 2021). The second mechanism involves the impairment of the intestinal mucosal barrier. Ubeda et al. discerned a downregulation of the FXR/SHP signaling pathway in the context of cirrhotic rats (Ubeda et al. 2016). Activation of the FXR pathway led to an augmentation in the expression of tight junction-related proteins and antimicrobial peptides, thereby preserving the integrity of the intestinal mucosal barrier and effectively ameliorating the progression of liver fibrosis (Sorribas et al. 2019). Meanwhile, Verbeke et al. observed a significant reduction in the expression of the FXR pathway in the ileum and jejunum in experimental BDL mice, resulting in the alteration of the intestinal mucosal barrier and the consequent translocation of gut microbiota. Conversely, FXR agonists enhanced the integrity of tight junctions and improved the intestinal mucosal barrier (Xiang et al. 2021). A recent investigation illuminated that Tropifexor (an FXR against) could elevate the expression of FGF19 within the ileum in BDL piglets, which enhanced the abundance of bile caid-biotransforming bacteria in the distal ileum, ameliorated intestinal barrier injury and suppressed BDL-induced liver injury, fibrosis, and ductular reaction (Xiao et al. 2021). These studies conveyed the pivotal role of BAs in shaping the gut microbiota and preserving the integrity of the intestinal mucosal barrier (Shi et al. 2022).

### The gut microbiota-bile acid axis as a target for CLD treatment

#### FXR ligands

Current studies have provided substantial evidence that multiple FXR agonists could ameliorate the gut microbiota, suppress BA synthesis and metabolism, and relieve liver fibrosis progression in animal models with cholestasis (Yan et al. 2021; Xiang et al. 2021). Currently, FXR agonists are in clinical trials for treating CLD (Table 2). Obeticholic acid, characterized as a potent FXR receptor activator, has completed phase II and III clinical trials on CLD in multiple studies (Kowdley et al. 2020; Trauner et al. 2019a, b). Remarkably, obeticholic acid exhibits the capability to attenuate serum hepatic enzyme levels and reduce bilirubin and alkaline phosphatase levels (ALP) in patients with PBC with inadequate response to or intolerance to ursodeoxycholic acid, suggesting long-term efficacy and safety in treating CLD. However, this drug has some adverse effects. The preeminent adverse effect was dose-dependent pruritus, thus demanding heightened attention in prospective research (Kowdley et al. 2020; Trauner et al. 2019a, b). Other types of FXR agonists, such as Cilofexor and Tropifexor, have also accomplished phase II clinical trials, which exhibited significant reduction in liver enzymes and gamma-glutamyl transferase (GGT) levels and possessed predictable pharmacokinetics and an acceptable safety-tolerability profile, thereby supporting further clinical exploration for FXR against in the treatment of CLD (Schramm et al. 2022; Trauner et al. 2019a). In addition, FGF19 acted as a downstream molecule after FXR aviation (Johansson et al. 2020). Using FGF19 analog to treat CLD could potently and robustly suppress hydrophobic BA levels and protect hepatocellular and intestinal epithelial from toxicity (Sanyal et al. 2021).

**Table 2:** The major clinical trial (phase 2 or higher) completed in CLD

Drug name	Compound	Trial phase	Indication	Clinical Trials No	Enrolled population	Study description	Clinical effects
Aldafermin (NGM282) (Sanyal et al. 2021)^)^	FGF19 mimetics	I	PSC	NCT02443116, NCT02704364	62	Evaluate the effects of the FGF19 analog on the circulating bile acid	FGF19 analog markedly reduced major hydrophobic BAs
Obeticholic (OCA) (Trauner et al. 2019a)	FXR ligands	III	PBC	NCT01473524	217	Evaluate long-term efficacy and safety of obeticholic acid in PSC	OCA improves serum liver enzyme, reduces total bilirubin concentrations, and has long-term efficacy and safety Adverse effects: pruritus, fatigue
Obeticholic (OCA) (Kowdley et al. 2020)	FXR ligands	I	PSC	NCT02177136	76	Investigate the efficacy and safety of OCA in PSC	OCA reduced serum ALP in PSC Adverse effects: mild to moderate dose-related pruritus
Cilofexor (GS-9674) (Trauner et al. 2019a)	FXR ligands	I	PBC	NCT02943460	52	Double-blind, placebo-controlled study evaluate the safety and efficacy of Cilofexor	Cilofexor significantly improves liver biochemistries and markers of cholestasis
Tropofexor (Schramm et al. 2022)	FXR ligands	I	PBC	NCT02516605	61	Double-blind, placebo-controlled study Evaluate the safety, tolerability, and efficacy of Tropofexor as a potential second-line therapy in PBC	Tropofexor improved cholestatic markers and has a predictable pharmacokinetics and safety-tolerability profile
Odevixibat (Thompson et al. 2022)	ASBT inhibitors	III	PFIC	NCT03566238	62	Evaluate the efficacy and safety of Odevixibat in PFIC	Odevixibat effectively reduced pruritus and serum BAs Adverse effects: diarrhea, frequent bowel movements
Odevixibat (Baumann et al. 2021)	ASBT inhibitors	I	Pediatric CLD	NCT02630875	8	Evaluate the safety, tolerability, and efficacy of Odevixibat pediatric CLD	Oral odevixibat was well tolerated, reduced serum BAs, and improved pruritus in pediatric CLD
Maralixibat (Gonzales et al. 2021)	ASBT inhibitors	I	ALGS	NCT02160782	31	Evaluate the safety and efficacy of maralixibat for children with ALGS	Maralixibat significantly reduced the level of serum BAs and improved the symptoms of pruritus Maralixibat is undergoing phase I clinical investigation for other types of CLD Adverse effects: gastrointestinal-related discomfort
Maralixibat (Loomes et al. 2022)	ASBT inhibitors	I	PFIC	NCT02057718	33	Open-label study Evaluate the efficacy and safety of Maralixibat in PFIC	Response to maralixibat was dependent on the progressive familial intrahepatic cholestasis subtype Some patients experienced rapid and sustained reductions in serum BAs and the symptoms of pruritus
Maralixibat (Bowlus et al. 2023)	ASBT inhibitors	I	PSC	NCT02061540	27	Open-label study Evaluated the safety and tolerability of Maralixibat in PSC	Maralixibat reduced bile acid levels in PSC and improved pruritus Adverse effects: gastrointestinal-related discomfort, diarrhea
Linerixibat (Levy et al. 2023)	ASBT inhibitors	I	PBC	NCT02966834	147	Evaluate dose-response, efficacy, and safety of Linerixibat in PBC	Linerixibat has no significant difference in pruritus versus placebo but was associated with a dose-dependent reduction in pruritus
Linerixibat (Hegade et al. 2017)	ASBT inhibitors	I	PBC	NCT01899703	22	Evaluate the efficacy and safety of PBC	Linerixibat was well tolerated without severe adverse events and effectively reduced pruritus severity Adverse effect: diarrhea
norUDCA (Fickert et al. 2017)	Norusodeoxycholid acid	I	PSC	NCT01755507	161	Evaluate the efficacy and safety in PSC	norUDCA reduced ALP levels in a dose-dependent manner with excellent safety

PSC: primary sclerosing cholangitis; PBC: primary biliary cholangitis; ALGS: Alagille syndrome; PFIC: progressive familial intrahepatic cholestasis; norUDCA: Norursodeoxycholid acid

#### ASBT inhibitors

ASBT inhibitor, characterized by suppressors of the ileal bile acid transport protein, could impede the hepatic-enteric circulation of BA and reduce serum BA concentration (Fuchs and Trauner 2022; Thibaut and Bindels 2022). Several ASBT inhibitor drugs, such as Odevixibat, Maralixibat, and Linerixibat, have been utilized in phase II and phase III clinical trials targeting pediatric CLD (Table 2) (Baumann et al. 2021; Bowlus et al. 2023; Gonzales et al. 2021; Hegade et al. 2017; Levy et al. 2023; Loomes et al. 2022; Thompson et al. 2022). ASBT inhibitors demonstrated the ability to reduce serum bile acid levels and alleviate pruritus markedly and represented a pioneering group of agents that manifest sustained and effective improvement in pediatric cholestasis (Gonzales et al. 2021). The most commonly observed adverse effects were associated with gastrointestinal disease, primarily self-limiting manifestations, such as diarrhea. Although ASBT inhibitors might become a novel therapeutic paradigm for CLD, further clinical studies are needed to observe the safety and efficacy of the drugs and expand the application of ASBT inhibitors in the realm of CLD (Gonzales et al. 2021; Levy et al. 2023).

#### Norursodeoxycholid acid

Since the 1980s, Norursodeoxycholid acid ** (**UDCA) has been shown to partially alleviate liver injury in PBC (Poupon et al. 1987). Subsequently, several studies have applied UDCA in a wide range of cholestatic disorders, making it a first-line drug for treating CLD (Beuers et al. 2015; Rost et al. 2004). However, long-term follow-up studies of UDCA in CLD have found that while UDCA might relieve the progression of liver cirrhosis and reduce the requirement for liver transplantation in some patients, approximately 40% of CLD patients are insensitive or irresponsive to UDCA treatment. Besides, no clear-cut survival benefit with UDCA in CLD has been displayed (Beuers et al. 2015; Cheung et al. 2019). 24-Norursodeoxycholid acid (norUDCA), a side chain shortened homolog of UDCA, presents several advantages over its parent compound: (a) norUDCA can be passively absorbed through cholangiocytes and undergo “choleheaptic shunting”, promoting HCO3-rich hypercholeresis, thus counteracting the toxic effects of BAs; (b) norUDCA has a stronger anti-inflammatory, antifibrotic and antiproliferative effect, directing regulating immune responses; (c) norUDCA is more hydrophilic and thereby less toxicity to hepatocytes and cholangiocytes (Fuchs and Trauner 2022; Beuers et al. 2015). Fickert et al. (Fickert et al. 2017) conducted a double-blind, randomized, multicenter study to evaluate the safety and efficacy of different doses of oral norUDCA in PSC patients. They found that norUDCA significantly reduced ALP levels in a dose-dependent manner with good safety. Phase III clinical trials are currently undergone to further evaluate the therapeutic efficacy.

#### Fecal microbial transplantation

Fecal microbial transplantation (FMT) refers to a procedure in which fecal matter, or stool is collected from a healthy donor, processed, and transplanted into the gastrointestinal tract of a recipient, aiming to improve the structure and function of the gut microbiota (Liu et al. 2024). Numerous studies have suggested that FMT might be a promising strategy for ulcerative colitis (Costello et al. 2019; Haifer et al. 2022). In CLD, alternations of gut microbiota are also common, and they can influence BAs metabolism through the gut-live axis. Therefore, FMT might have potential therapeutic effects in the treatment of CLD (Fang et al. 2022; Gerussi et al. 2020). Allegretti et al. (2019) conducted a pilot clinical trial of FMT for treating PSC and found the diversity of gut microbiota increased post-FMT. Additionally, 30% of patients experienced a ≥ 50% decrease in ALP levels with no related adverse events, which suggested that FMT might be safe for treating PSC. However, the safety and efficacy of FMT in treating CLD require further evaluation through larger prospective, multicenter clinical trials.

## Conclusion

In summary, the interaction between gut microbiota and BA defies unidirectionality. BAs can mold the gut microbiota; conversely, the gut microbiota can alter BA composition. This bidirectional dynamic, a reciprocal interplay, assumes a pivotal role in CLD. In CLD, the elevated BA concentrations within the liver can incite inflammatory cascades, thereby instigating hepatocyte death and effectuating the progression of liver fibrosis. In addition, impediments of bile flow into the intestines can incite gut microbiota dysbiosis, culminating in the disruption of the intestinal mucosal barrier and microbial translocation. Bacteria translocation can trigger immune response and propel the progression of CLD. Currently, clinical trials are investigating pharmaceutical agents that target the gut microbiota-bile acid axis. However, given the potential side effects and the possible lack of specificity to certain tissues or cell types, there is a clear necessity for further research to evaluate the safety and efficacy of these drugs in clinical practice. Moreover, there is an urgent need for a deeper understanding of the mechanisms of the gut microbiota-bile acid axis in CLD for exploring potential therapeutic targets.

## Data Availability

All data generated or analyzed during this study are included in this manuscript.

## Abbreviations

CLD: Cholestatic liver diseases
Bas: Bile acids
PBC: Primary biliary cholangitis
PSC: Primary sclerosing cholangitis
PFIC: Progressive familial intrahepatic cholestasis
DCs: Dendritic cells
TGR5: Takeda G protein-coupled receptor 5
ALP: Alkaline phosphatase
GGT: Gamma-glutamyl transpeptidase
HC: Healthy control
IBD: Inflammatory bowel disease
TJ: Tight junction
KC: Kupffer cells
HSCs: Hepatic stellate cells
PAMPs: Pathogen-associated molecular patterns
LPS: Lipopolysaccharide
LTA: Lipoteichoic acid
TLR: Toll-like receptor
NLR: Domain-like receptor
IgA: Infection-free globule
BSEP: Bile salt export pump
SCFAs: Short-chain fatty acids
NTCP: Sodium taurocholate co-transporting polypeptide
OATPs: Organic anion-transporting polypeptides
FGF19: Fibroblast growth factor 19
FGFR4: Fibroblast growth factor receptor 4
ASBT: Apical sodium-dependent BA transporter
IBABP: Ileal bile acid-binding protein
OSTα/β: Organic solute transporter-α/β
LXR: Liver X receptor
S1PR2: Sphingosine-1-phosphate receptor 2
ZBP1: Z-DNA-binging protein 1
p-MLKL: Phosphorylated-mixed lineage kinase domain-like pseudokinase
CYP7A1: Cholesterol 7α-hydroxylase
GDCA: Conjugated BA glycodeoxycholate
EGF: Epidermal growth factor
VEGF: Vascular endothelial growth factor
TNFα: Tumor necrosis factor
DR: Ductular reaction
GPCR: G-protein-linked receptor
cAMP: Adenosine monophosphate
NLRP3: NOD-like receptor protein 3
DCA: Deoxycholic acid
LCA: Lithocholic acid
BSH: Bile salt hydrolase
CDCA: Chenodeoxycholic acid
NUDCA: Norursodeoxycholid acid
norUDCA: 24-Norursodeoxycholid acid
FMT: Fecal microbial transplantation
